# Neuronal activity regulates remyelination via glutamate signalling to oligodendrocyte progenitors

**DOI:** 10.1038/ncomms9518

**Published:** 2015-10-06

**Authors:** Hélène O. B. Gautier, Kimberley A. Evans, Katrin Volbracht, Rachel James, Sergey Sitnikov, Iben Lundgaard, Fiona James, Cristina Lao-Peregrin, Richard Reynolds, Robin J. M. Franklin, Ragnhildur T Káradóttir

**Affiliations:** 1 Wellcome Trust-Medical Research Council Cambridge Stem Cell Institute and Department of Veterinary Medicine, University of Cambridge, Tennis Court Road, Cambridge CB2 1QR, UK; 2 Faculty of Medicine, Division of Brain Sciences, Imperial College London, Hammersmith Hospital, London W12 0NN, UK; 3 Wellcome Trust-Medical Research Council Cambridge Stem Cell Institute and Department of Clinical Neurosciences, University of Cambridge, Cambridge CB2 0AH, UK

## Abstract

Myelin regeneration can occur spontaneously in demyelinating diseases such as multiple sclerosis (MS). However, the underlying mechanisms and causes of its frequent failure remain incompletely understood. Here we show, using an *in-vivo* remyelination model, that demyelinated axons are electrically active and generate *de novo* synapses with recruited oligodendrocyte progenitor cells (OPCs), which, early after lesion induction, sense neuronal activity by expressing AMPA (α-amino-3-hydroxy-5-methyl-4-isoxazolepropionic acid)/kainate receptors. Blocking neuronal activity, axonal vesicular release or AMPA receptors in demyelinated lesions results in reduced remyelination. In the absence of neuronal activity there is a ∼6-fold increase in OPC number within the lesions and a reduced proportion of differentiated oligodendrocytes. These findings reveal that neuronal activity and release of glutamate instruct OPCs to differentiate into new myelinating oligodendrocytes that recover lost function. Co-localization of OPCs with the presynaptic protein VGluT2 in MS lesions implies that this mechanism may provide novel targets to therapeutically enhance remyelination.

## Abstract (web summary)

Myelin regeneration can occur spontaneously in demyelinating diseases but the underlying mechanisms are incompletely understood. Here the authors show that neuronal activity and glutamatergic synapses instruct oligodendrocyte progenitor cells to differentiate into new myelinating oligodendrocytes.

## Graphical abstract

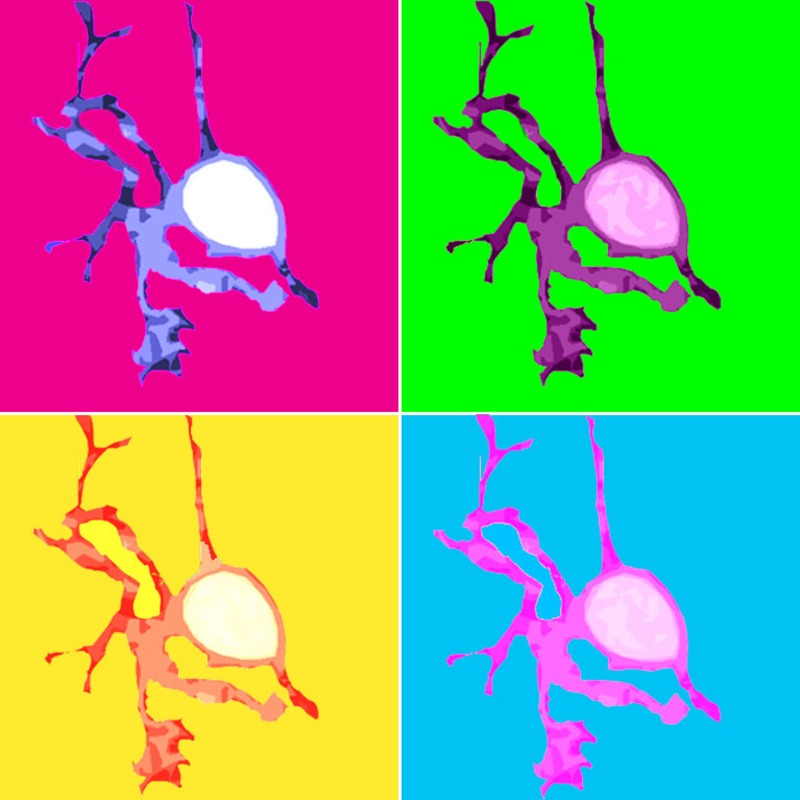

Remyelination of the central nervous system (CNS) axons is essential for functional recovery after demyelinating injuries. It can occur as a spontaneous regenerative process in multiple sclerosis (MS) and other neurological conditions, including spinal cord injury. Oligodendrocyte progenitor cells (OPCs), which comprise ∼5% of all cells in the adult CNS and are the principal proliferative cell type^1^, respond to demyelinating injury by differentiating into myelinating oligodendrocytes to restore function^2^,^3^. This regenerative process is controlled by a precisely orchestrated but still incompletely understood array of signalling events^4^. However, remyelination often fails, primarily owing to failure of OPC lineage progression rather than a depletion of OPCs, many of which persist in chronic demyelinated lesions^5^. Thus, many attempts at promoting remyelination therapeutically have focused on the control of OPC differentiation. A critical feature of the injury environment that has been largely overlooked is the demyelinated axon and the role it might play in regulating OPC lineage progression during remyelination.

In development, white matter OPCs express glutamate receptors^6^,^7^ and receive synaptic input^6^,^8^,^9^,^10^,^11^ from unmyelinated axons^9^,^10^, enabling them to monitor and respond to neuronal activity. Both the synaptic input and glutamate signalling regulate OPC proliferation^11^,^12^ and myelination^13^,^14^,^15^,^16^, although neuronal activity is not essential for myelination^13^,^17^. In demyelinated lesions of the corpus callosum OPCs and progenitor cells recruited from the sub-ependymal zone receive synaptic input from axons^18^,^19^. However, it is unclear whether this input is from unmyelinated corpus callosal axons, as up to 70% of callosal axons are unmyelinated^20^, or from demyelinated axons that generate *de novo* synapses to recruited OPCs. Even less is known of the effects of such signalling on remyelination. Moreover, it is uncertain of the extent to which glutamate exacerbates demyelination via excitotoxicity^21^,^22^ or promotes remyelination^13^,^23^.

Here we show that demyelinated axons generate synaptic contacts to OPCs, and that neuronal activity regulates remyelination, by synaptic release of glutamate, instructing OPCs to differentiate into new myelinating oligodendrocytes.

## Results

### The toxin-induced demyelination model

**Figure 1:**
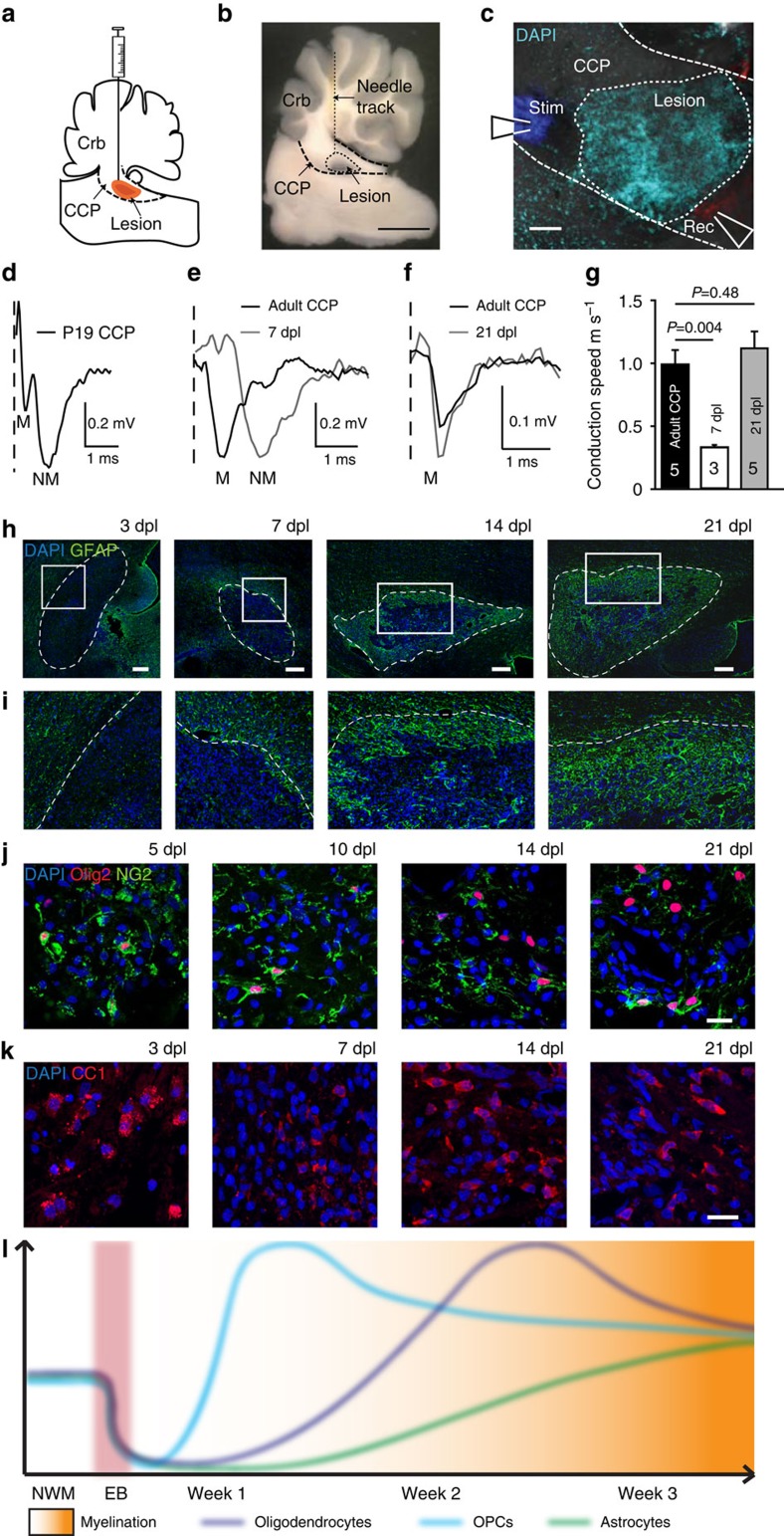
The toxin-induced demyelination model. (**a**) Demyelinated lesions were created by injection of 0.01% EB into the rat CCP (Crb, cerebellum). (**b**) Acute cerebellum and brainstem section containing a lesion in the CCP (delineated by dashed lines). The needle track going through the cerebellum is visible on the left of the vertical dashed line. Scale bar, 2 mm. (**c**) Fixed section after compound action potential recording, the lesion (delineated by dashed lines) is visible with 4,6-diamidino-2-phenylindole (DAPI) staining due to increased macrophages and OPC infiltration; the location of stimulating (stim, dark blue DiD) and recording (rec, red DiI) electrodes are marked. Scale bar, 200 μm. (**d**–**f**) TTX-subtracted compound action potential recordings. (**d**) At p19, peaks for both myelinated (M) and non-myelinated axons (NM) were detected. (**e**,**g**) At 7 dpl, demyelinated axons (grey trace) have a peak latency similar to non-myelinated axons, whereas (**f**,**g**) when remyelinated at 21 dpl (grey trace) the peak latency is similar to adult myelinated axons (black trace). Numbers of brain slices are shown on bars. Data represent means±s.e.m. The *P*-value is from Holm–Bonferroni *post-hoc* test after a one-way ANOVA (*P=*0.003). (**h**) Timeline of GFAP expression in CCP lesions at 3, 7, 14 and 21 dpl; scale bar, 200 μm, (**i**) Magnified images from **h** of the regions indicated by the white box. (**j**) Timeline of oligodendrocyte lineage cells in the lesion, with Olig2^+^ and NG2^+^ OPCs, and (**k**) CC1^+^ oligodendrocytes. Scale bars, 20 μm. (**l**) Diagram showing the peak appearance of oligodendrocytes, OPCs and astrocytes in the CCP following EB lesion. The red shaded area marks the timing of the EB injection and the onset of demyelination with the associated death of all nucleated cell types within the lesion. Re-colonization of the lesion occurs as follows: OPCs arrive in the lesion and proliferate before differentiating into oligodendrocytes. Astrocytes start to repopulate the lesion in week 2. The orange shading indicates the remyelination process; darker orange corresponds to more remyelination.

To investigate the signalling between demyelinated axons and OPCs within a demyelinated area, we used a toxin-induced focal demyelinating lesion (Fig. 1a,b). In this model, there is little axonal pathology and remyelination proceeds to completion with a clear temporal separation between the acute demyelination phase and the subsequent stages of remyelination^24^,^25^. Moreover, the adult rat caudal cerebellar peduncle (CCP) is fully myelinated at the time of toxin injection; thus, all bare axons are demyelinated axons (Supplementary Fig. 1 and see Methods)^24^.

First, we addressed whether demyelinated axons in the CCP conduct action potentials (Fig. 1c–g). During developmental myelination of the CCP, there is a clear separation between the conduction speed of myelinated and unmyelinated axons (Fig. 1d). However, at 3 months of age, at the time of lesion induction, the latency peak associated with conduction in unmyelinated axons was undetectable (Fig. 1e, black trace), indicating that all CCP axons are myelinated at this time. To determine whether the demyelinated axons can still conduct, we recorded from the lesions at 7 days post lesion (dpl), at the time when OPC recruitment is at its maximum^25^ (Fig. 1j,l). We found that demyelinated axons conducted action potentials with a speed (0.29±0.04 m s^−1^, at room temperature) similar to that of unmyelinated axons during development (0.45 m s^−1^ at p19; Fig. 1d,e), thus demonstrating that all the axons within the focal CCP lesion are demyelinated but, most importantly, are capable of impulse conduction. Recovery of fast action potential propagation was established by 21 dpl, when remyelination is largely complete (Fig. 1f,g), with conduction speeds identical to those of the pre-lesion adult CCP (1.12±0.13 versus 0.99±0.12 m s^−1^, respectively; *P=*0.48, one-way analysis of variance (ANOVA) followed by Holm–Bonferroni test, *n*=5) and significantly faster than at 7 dpl (*P=*0.008, one-way ANOVA followed by Holm–Bonferroni test, *n*=3). Therefore, we have clearly established that in this lesion model demyelinated axons can conduct, with the speed of unmyelinated axons, and there is a full functional recovery of conduction speed on remyelination. Although remyelination generates thinner myelin than developmental myelination^26^, the resulting internodes are shorter^27^, which, along with other changes such as nodal geometry and voltage-gated sodium channel density, may compensate for the thinner myelin^28^ and restore the conduction velocity to its normal value^29^.

Second, we investigated the cellular profile of the demyelinating lesion as it progresses from demyelination to remyelination. Ethidium bromide (EB) is cytotoxic and will damage all nucleated cell types but leaves the axons intact (Supplementary Fig. 1 and see Methods)^24^; thus, all cells within the repairing lesion are recruited from surrounding tissue. As previously demonstrated for early timepoints^24^, we found that the lesions lack astrocytes (Fig. 1h,i,l); however, at 14 dpl astrocytes emerge from the lesion edge and at 21 dpl they have fully re-colonized the lesion, but appear slightly more immunoreactive to glial fibrillary acidic protein (GFAP) than in the adjacent normal-appearing white matter. OPCs (NG2^+^ and Olig2^+^) are present at 3 dpl^25^,^30^ and reach a much higher density than in normal-appearing adult white matter at 5–7 dpl (Fig. 1j,l), but by 21 dpl their numbers have declined to near-normal adult white matter levels. Staining for CC1, a marker of differentiated oligodendrocytes, at 3 dpl is punctate/globular and is likely to be the postdemyelination remnants of antigen present within phagocytic macrophages. We detected few differentiated oligodendrocytes (CC1^+^) at 7 dpl (Fig. 1k,l), but their numbers overshot at 14 dpl before decreasing to reach a density similar to that in normal-appearing white matter at 21 dpl (Fig. 1k,l). This indicates that there is an overproduction of newly formed oligodendrocytes (CC1^+^) that presumably never reach targets and die, similar to as occurs during development^31^.

Together, these data reveal that the lesion provides an excellent model to study the interactions between demyelinated axons and OPCs *in vivo*, because at early timepoints, it lacks astrocytes and is only populated by OPCs and macrophages; all OPCs within the lesion have been recruited from surrounding areas; the axons are healthy and electrically active; and all axons lacking myelin within the lesion were previously myelinated.

### Recruited OPCs express glutamate receptors

**Figure 2:**
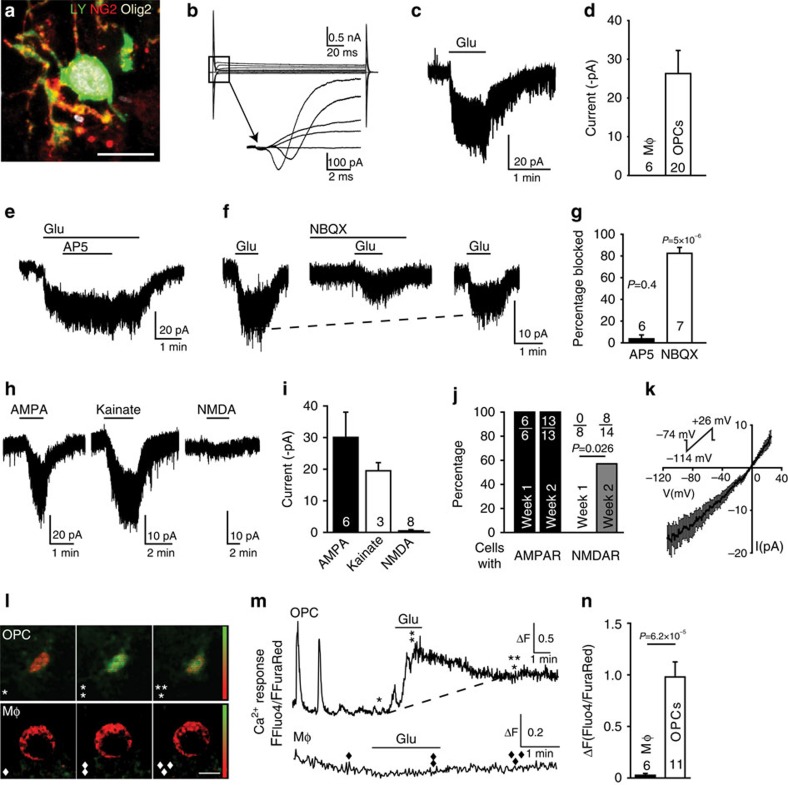
Glutamate signalling in remyelinating lesions. (**a**) Voltage-clamped OPC, filled with lucifer yellow (LY) and identified after recording by labelling for NG2 and Olig2 (scale bar, 10 μm) (**b**) with voltage-gated sodium currents (20 mV steps from −74 mV; inset: leak subtracted trace). (**c**) Response of a recruited OPC to 100 μM glutamate (Glu). (**d**) Mean peak current for glutamate-evoked currents in recruited OPCs (a specimen trace shown in **c**) and macrophages (**e**–**g**). The glutamate-evoked current is unaffected by 200 μM AP5 (*P*=0.4, *n*=6) but inhibited by 25 μM NBQX (*P*=5 × 10^−6^, *n*=7, one sample *t*-test). (**h**,**i**) AMPA (20 μM), kainate (30 μM) and NMDA (60 μM) evoked inward currents in OPCs. (**j**) Proportion of OPCs with AMPA-evoked currents (in the first week post lesion, 6 out of 6 cells recorded showed AMPA-evoked currents; in the second week, 13 out of 13 cells responded to AMPA) and NMDA-evoked currents; NMDA-evoked currents only became detectable in OPCs at the start of the second week post lesion induction, *P*=0.026 (*χ*^2^-test with Yates correction, compared with the first week; in the first week, 0 out of 8 cells recorded had detectable NMDA-evoked currents; for the second week, 8 out of 14 had detectable NMDA receptor responses). (**k**) Current–voltage relationship (voltage ramp from −134 to +26 mV) for glutamate-evoked current in OPCs (*n*=7). (**l**–**n**) Glutamate-evoked [Ca^2+^]_i_ rises in OPCs but not in macrophages (*P*=6.2 × 10^−5^, Welch’s corrected unpaired *t*-test, *n*=11 and 6 cells, respectively, from 3 independent experiments) measured by taking the fluorescent intensity ratio of Fluo-4/FuraRed (low Ca^2+^: red; high Ca^2+^: green). Scale bar, 10 μm. Numbers of cells are indicated on bars, data represent means±s.e.m. in **d**,**g**,**i**,**n**.

To test whether adult OPCs recruited to areas of demyelination can sense glutamate released from active axons, as they do during development, we voltage-clamped OPCs within the lesion at 5–8 dpl in acute brain slices containing the lesion (Fig. 1b). OPCs were identified by their dye-filled morphology, electrophysiological properties and by post-recording immunohistochemistry^8^ (Fig. 2a,b and see Methods). OPCs were both morphologically and electrophysiologically distinct from macrophages (Supplementary Fig. 2), which are large circular cells that lack voltage-gated sodium currents typical of OPCs^8^ but have a slowly activating outward current at potentials above 40 mV, characteristic of microglia^32^,^33^ (Supplementary Fig. 2b,d). Despite a small proportion of macrophages expressing NG2, we never observed co-expression of Olig2 in these cells (Supplementary Fig. 2e). Glutamate evoked an inward current in NG2^+^ and Olig2^+^ OPCs (100 μM, 25.1±5.8 pA at −74 mV; Fig. 2c,d) but not in macrophages (Fig. 2d and Supplementary Fig. 2c). The glutamate-evoked current in NG2^+^ and Olig2^+^ OPCs was reduced by the selective AMPA (2-amino-3-(3-hydroxy-5-methyl-isoxazol-4-yl)propanoic acid)/kainate receptor antagonist NBQX (2,3-dihydroxy-6-nitro-7-sulfamoyl-benzo[f]quinoxaline-2,3-dione; 25 μM)^34^, but not the selective NMDA (*N*-methyl-D-aspartate) receptor antagonist D-AP5 (D(-)-2-amino-5-phosphonovaleric acid; 200 μM)^34^ (Fig. 2e–g; *P*=5 × 10^−6^ and *P*=0.4, respectively, one sample *t*-test, *n*=7 and 6, respectively). Similarly, both AMPA (20 μM) and kainate (30 μM), but not NMDA (60 μM), evoked an inward current in OPCs (Fig. 2h,i). However, at the beginning of the second week post lesioning, NMDA-evoked currents in OPCs could be detected (Fig. 2j; NMDA currents were detected in 0 out of 8 OPCs in the first week but in 8 out of 14 OPCs in the second week; *P*=0.026, *χ*^2^-test with Yates correction), along with AMPA receptor currents (detected in 6 out of 6 OPCs in the first week and in 13 out of 13 OPCs in the second week). This suggests that, at the time of recruitment (the first week post lesioning), OPCs in the lesions mainly express AMPA and kainate receptors, making them capable of sensing glutamate released from active neurons.

The positive rectification of glutamate-evoked currents in the recruited OPCs varied, with the rectification index [(*I*_+20mV_−*I*_rev_)/(*I*_rev_−*I*_−70mV_)] ranging from 0.18 to 2 (Fig. 2k), indicative of OPCs expressing a variable mixture of non-calcium-permeable and calcium-permeable AMPA receptors^35^. Glutamate (100 μM) elicited a rise in intracellular calcium concentration ([Ca^2+^]_i_) in ∼60% of OPCs (11 out of 18 cells) in the lesions (Fig. 2l–n and Supplementary Fig. 3d,e) and only in these cells were spontaneous [Ca^2+^]_i_ spikes detected (Fig. 2m). No [Ca^2+^]_i_ changes or glutamate-evoked responses were detected in macrophages (*P=*6 × 10^−5^, Welch’s corrected unpaired *t*-test, *n*=6 for macrophages, *n*=13 for OPCs; Fig. 2l–n) or demyelinated axons (Supplementary Fig. 3d,e). Given that at the time of OPC recruitment the lesion is devoid of astrocyte cell bodies^24^ (Fig. 1h,i,l), these data indicate that at this timepoint the OPCs are the only cells within the lesion capable of responding to glutamate.

### Demyelinated axons generate *de novo* synapses with OPCs

**Figure 3:**
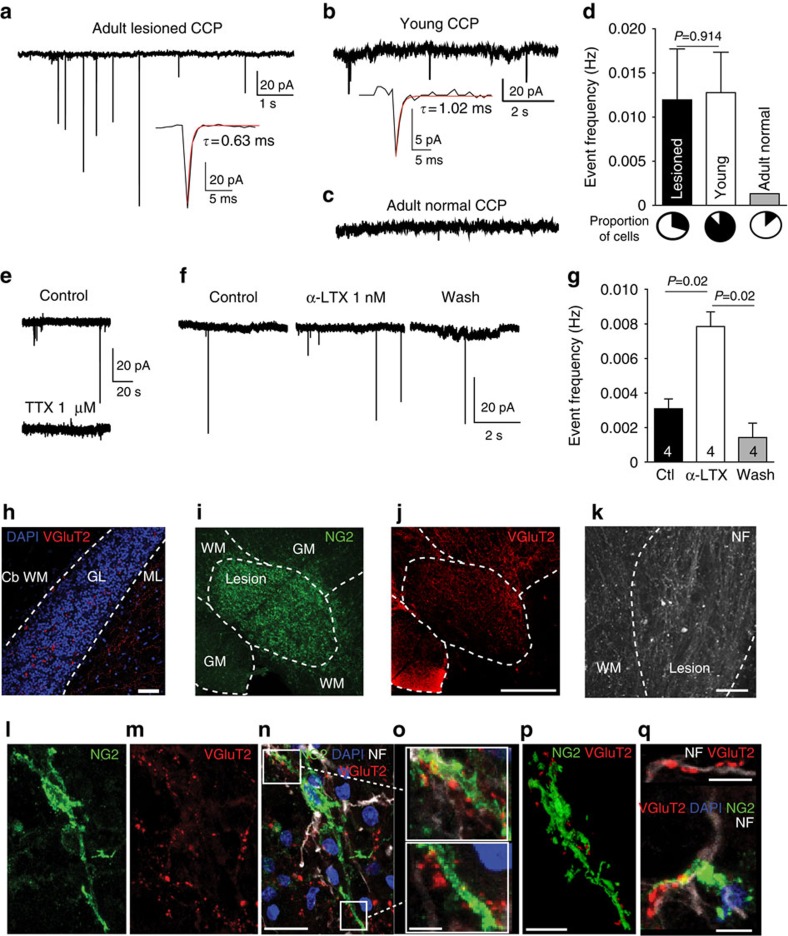
OPCs in remyelinating lesions receive synaptic inputs. (**a**) Recruited OPCs receive synaptic input from demyelinated axons (15/45 cells; inset: specimen of a fitted synaptic event), similar to (**b**) OPCs at the start of developmental myelination in the CCP of NG2-EYFP knock-in mice (seven out of eight cells recorded; inset: specimen of a fitted synaptic event), whereas (**c**) after myelination of the CCP is complete OPCs barely receive synaptic inputs (one out of eight cells recorded; *P=*0.01, *χ*^2^-test with Yates correction compared with young CCP). (**d**) Average frequency of detected synaptic input in OPCs in lesion (5–8 dpl), in the CCP at the start of myelination (p5–8 NG2-EYFP mice) and once myelination is complete (p98–105 NG2-EYFP mice). Data are means±s.e.m., significance tested by unpaired *t*-test, *n*=6. The circles underneath the bar graph depict the proportion of OPCs with (black) and without (white) synaptic inputs. (**e**) The synaptic inputs are blocked by 1 μM TTX. (**f**,**g**) α-Latrotoxin (α-LTX; 1 nM) increases the frequency of synaptic events detected in OPCs (*P*=0.02, paired Student’s *t*-test, all cell numbers are indicated on each bar graph, data are means±s.e.m.). (**h**) The presynaptic marker VGluT2 is present in the adult cerebellar grey matter (GM), but not in normal white matter (WM). Scale bar, 50 μm. (**i**,**j**) At 7 dpl, NG2 and VGluT2 staining are increased in demyelinating lesions. Scale bar, 500 μm. (**k**) Neurofilament (NF) is equally detectable in normal and lesioned white matter. Scale bar, 200 μm. (**l**–**n**) OPCs establish synapses with demyelinated axons. Scale bar, 20 μm. (**o**) Magnifications of the cell in **l**–**n**. Scale bar, 5 μm. (**p**) Iso-surface 3D deconvolution of the cell in **l**–**n** generated using Imaris software, showing synapses located on the OPC processes. Scale bar, 20 μm. (**q**) Top: synaptic vesicles along a demyelinated axon; bottom: OPC extending processes towards VGluT2 vesicles. Scale bars, 5 μm.

During development, white matter OPCs receive synaptic inputs^6^,^8^,^9^,^10^ from unmyelinated axons^9^,^10^, via postsynaptic AMPA receptors^36^, and after demyelinating injury corpus callosal axons generate synapses with recruited sub-ependymal zone progenitors^18^ and OPCs^19^. However, as the majority of corpus callosal axons are unmyelinated, we assessed whether *bona fide* demyelinated axons are capable of regenerating synaptic inputs to OPCs by voltage clamping OPCs within the EB lesion, where all axons were previously myelinated. We detected spontaneous excitatory synaptic currents (*τ*_decay_=0.78±0.03 ms, *n*=15; Fig. 3a,d; in some OPCs, outward currents were also detected, presumably mediated by GABAergic input^8^), reminiscent of the EPSCs detected at a similar frequency (*P*=0.91, unpaired *t*-test, *n*=6) in OPCs in the mouse CCP at p7, at the start of myelination^37^ (Fig. 3b,d). In contrast, when myelination was complete, in 3-month-old mice, synaptic inputs were barely detected (in 1 out of 8 cells, a single event above noise levels was detected compared with 7 out of 8 cells in young CCP with multiple events per cell; *P*=0.01, *χ*^2^-test with Yates correction; Fig. 3b–d) and the total frequency of synaptic events detected when myelination was complete was 0.16±0.16 mHz compared with 10.8±3.7 mHz during development (*P*=0.012, unpaired *t*-test, *n*=8), indicating that OPCs receive relatively few, if any, synaptic inputs in a white matter tract that is fully myelinated. The events detected in lesions were abolished by blocking action potentials with 1 μM tetrodotoxin (TTX; Fig. 3e) and increased in frequency with 1 nM α-latrotoxin, which, as expected for this concentration^38^, promoted presynaptic neurotransmitter release by ∼2.5-fold (3.1±0.6 versus 7.8±0.8 mHz; *P=*0.02, paired *t*-test, *n*=4; Fig. 3f,g)^10^. The pharmacological properties and the fast kinetics of these inputs are identical to those of the synaptic inputs that OPCs receive from unmyelinated axons^9^,^10^ and differ greatly from what would be expected if generated by reversed cycling of glutamate transporters^10^,^39^. Thus, these results indicate the existence of defined synaptic structures between recruited OPCs and demyelinated axons, which form after demyelination.

To examine the pattern of expression of presynaptic terminals within demyelinated lesions, we labelled for VGluT2, a marker for glutamatergic presynaptic release sites known to be expressed by neurons whose axons pass through the CCP^40^. In the demyelinated lesion, there was clear punctate labelling for VGluT2 (observed for seven lesions). In contrast, VGluT2 labelling was undetectable within the nearby normal-appearing white matter (Fig. 3j) and in the adult white matter of unlesioned animals (Fig. 3h). The punctate VGluT2 labelling seen in the lesion was similar to that seen in the grey matter (Fig. 3h,j). The intensity of neurofilament labelling was unchanged between the lesion and normal white matter areas (Fig. 3k), demonstrating equal detection of intra-axonal proteins between myelinated and demyelinated fibres. Similar to what occurs for unmyelinated axons during development, presynaptic formations occurred along the demyelinated axons independent of OPC proximity^9^ (Fig. 3q, top panel), although strikingly, OPC processes seemed to be preferentially located near VGluT2 puncta on the demyelinated axons (Fig. 3l–q; observed for 196 NG2^+^ cells with an average of 10±2 VGluT2 puncta per cell). Together, these data strongly suggest that demyelinated axons establish *de novo* synapses with recruited OPCs.

### Synaptic transmission regulates remyelination

**Figure 4:**
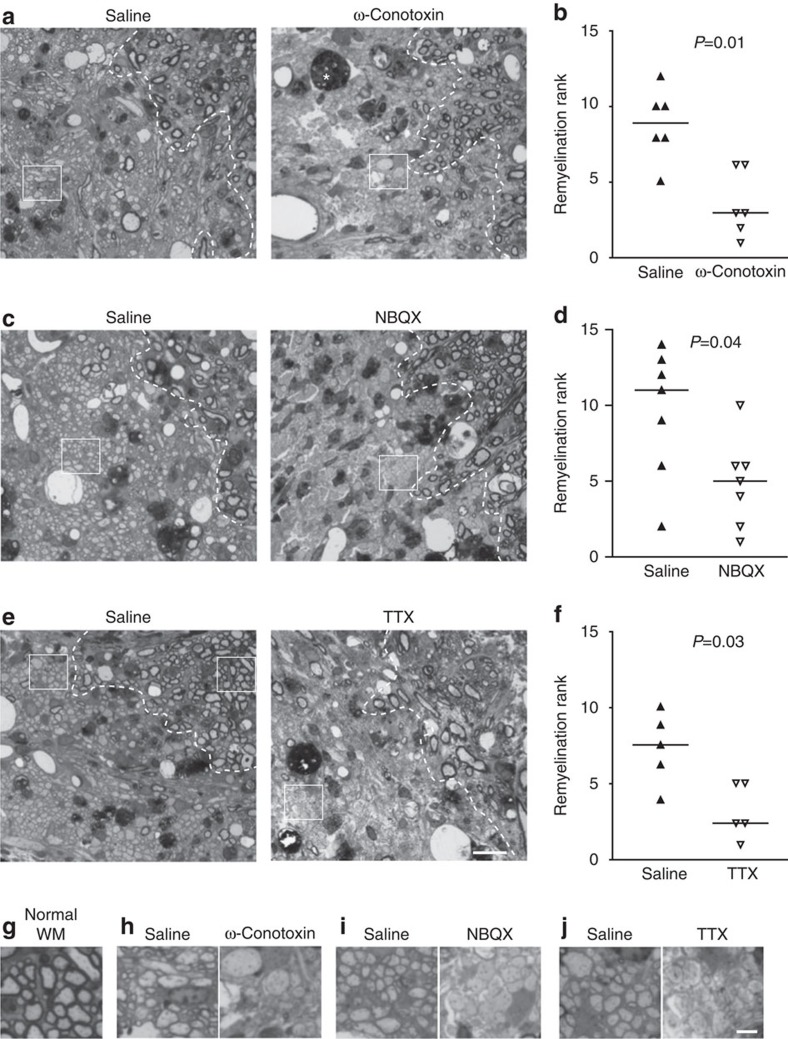
Remyelination is dependent on neuronal activity. Toluidine blue-stained semi-thin sections of CCP 21 dpl, infused with saline, or from 3 dpl (**a**), ω-conotoxin (11 μM; blocking neurotransmitter vesicular release), (**c**) NBQX (250 μM; blocking AMPA/kainate receptors), or (**e**) TTX (50 nM) (scale bar, 20 μm) and (**b**,**d**,**f**) scored by blinded ranking analysis; higher ranks represent more remyelination, each symbol represents one animal, *P*-values are from Mann–Whitney *U*-test. (**g**–**j**) High-magnification images of (**g**) normal-appearing white matter and (**h**–**j**) saline (left), and (**h**) ω-conotoxin-, (**i**) NBQX- and (**j**) TTX (right)-infused lesion. Scale bar, 5 μm.

To examine the functional importance of the synaptic input from the demyelinated axons to the OPCs, we infused either 0.9% saline or an antagonist to specifically block distinct stages of synaptic transmission into the lesion via an intracerebral cannula, connected to an osmotic minipump. Infusions into the lesion were from 3 dpl, when OPCs are being recruited, to 21 dpl^25^ (see Methods and Fig. 1j,l). We have previously shown that the delivery is local to the lesion, as infusing dye into the lesion via osmotic minipump results in the dye being confined within the lesion^41^. First, we infused 11 μM ω-conotoxin MVIIC, a selective N-, P- and Q-type calcium channel blocker^42^, to block vesicular release of neurotransmitter, from 3 dpl. As the lesion is devoid of astrocyte cell bodies^24^ (Fig. 1h,i,l), apart from axons only OPCs and (monocyte- and microglia-derived) macrophages are present and neither express functional N-, P- and Q-type voltage-gated calcium channels^43^,^44^; thus, we attribute the effects of ω-conotoxin MVIIC to its action on demyelinated axons. Blinded ranking analysis of remyelination, an established histological analysis that assesses cross-sections of the entire lesion, revealed that blocking vesicular neurotransmitter release significantly impaired remyelination compared with saline controls (*P=*0.01, Mann–Whitney rank analysis, *n*=6 per group; Fig. 4a,b,h), with fewer axons remyelinated (saline: 63±4%, ω-conotoxin MVIIC: 23±5%; *P=*5 × 10^−4^, unpaired *t*-test, *n*=6), but had no effect on lesion size (saline: 0.53±0.05 mm^2^, ω-conotoxin MVIIC: 0.53±0.05 mm^2^; *P*=0.97, unpaired *t*-test, *n*=6 per group). This indicates that blocking vesicular release of neurotransmitter reduces remyelination, similar to that during development for myelination^15^,^16^.

Second, we blocked AMPA/kainate receptors, which are expressed on the OPCs (Fig. 2) and are mainly located postsynaptically at the axon-OPC synapse^36^, by infusing NBQX from 3 dpl (250 μM; Fig. 4c,d,i), a selective AMPA/kainate receptor antagonist that does not affect NMDA and metabotropic glutamate receptors^34^, into the lesion. As macrophages do not respond to glutamate (Fig. 2d,l–n and Supplementary Fig. 2c) and demyelinated axons have not been reported to express AMPA receptors (nor did we detect glutamate-evoked calcium changes in demyelinated axons), we attribute the action of NBQX to be on OPCs, presumably at the postsynaptic site. Blocking AMPA/kainate receptors reduced remyelination (*P*=0.039, Mann–Whitney rank analysis, *n*=7 per group) without affecting lesion size (0.52±0.06 mm^2^ (saline) versus 0.66±0.08 mm^2^ (NBQX); *P*=0.20, unpaired *t*-test, *n*=7 per group).

**Figure 5:**
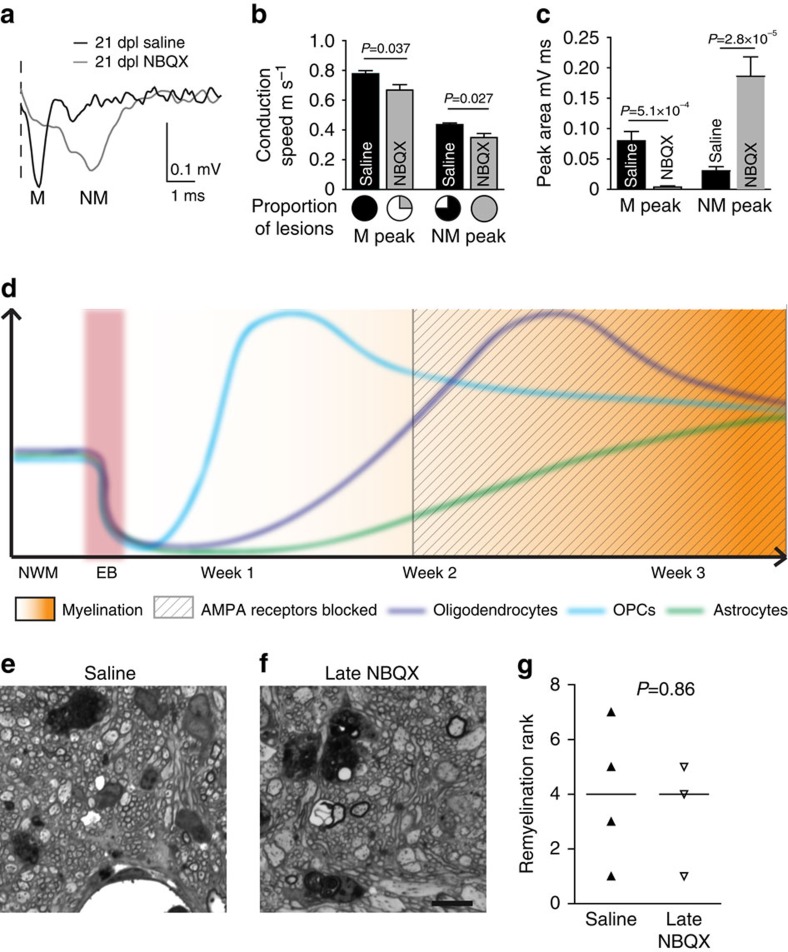
Remyelination depends on activation of AMPA receptors within the lesion at early timepoints. (**a**) TTX-subtracted compound action potential recordings from saline (black trace)- and NBQX-treated lesions from 21 dpl (grey trace). (**b**) Average conduction speed for the latency peak associated with myelinated axons (M peak) and non-myelinated axons (NM), for saline- and NBQX-treated samples (data from four slices for each condition). Circles underneath depict the proportion of recorded lesions with the latency peak detected (white fraction: no detection of peak; black/grey: proportion of lesion where the latency peak is detected in saline/NBQX-treated lesions). (**c**) The average latency peak area reflects the number of axons contributing to each peak. It is noteworthy that there are fewer axons contributing to the latency peak associated with myelinated axons and far more contributing to the peak associated with demyelinated axons in NBQX-treated samples (*P*-values are from unpaired *t*-test). (**d**) The timeline diagram of the lesion remyelination process (Fig. 1l), shaded with grey box to indicate when NBQX infused at later timepoints is present. (**e**) Toluidine blue-stained semi-thin sections of CCP 21 dpl, infused with saline or (**f**) NBQX (250 μM; blocking AMPA/kainate receptors) from 10 dpl (scale bar, 10 μm) (**g**) scored by blinded ranking analysis; higher ranks represent more remyelination, each symbol represents one animal, *P*-values from Mann–Whitney *U*-test.

This reduction in remyelination significantly affected conduction speed through the lesion. We detected latency peaks associated with conduction of myelinated fibres in all lesions infused with saline but detected only in 25% of lesions infused with NBQX (Fig. 5b), which had a slightly slower conduction speed (0.78±0.02 m s^−1^ (saline) versus 0.67±0.02 m s^−1^ (NBQX); *P=*0.037, unpaired *t*-test, *n*=4). The majority of axons in NBQX-treated lesions were demyelinated, as 100% of lesions had a latency peak associated with demyelinated axons (a conduction speed of 0.34±0.02 m s^−1^) compared with 75% of all saline lesions (a conduction speed of 0.43±0.01 m s^−1^; *P*=0.027, unpaired *t*-test, *n*=4; Fig. 5b). The reduced recovery of saline-treated lesions indicates that saline dilutes some essential factors for remyelination, as a full recovery of the action potential propagation is not fully established by 21 dpl in saline, unlike in uninfused lesions (Fig. 1f,g). Nevertheless, the demyelinated latency peak in NBQX-treated lesion had a far larger area (0.03±0.00 mV*ms (saline) versus 0.19±0.03 mV*ms (NBQX); *P*=2.8 × 10^−5^, unpaired *t*-test, *n*=4). The peak area reflects the activity of all the stimulated axons and is thus directly proportionate to the number of demyelinated axons, indicating that there are many more demyelinated axons in NBQX-treated samples. Together, these results demonstrate that AMPA/kainate receptors are important for efficient remyelination.

Previously, we have shown that blocking NMDA receptors with MK-801, a highly selective open channel NMDA receptor antagonist^34^, reduces remyelination^13^, similar to blocking AMPA receptors or vesicular release of glutamate. As we only detect NMDA receptor expression in OPCs at the second week post lesioning (Fig. 2j), at a time when OPCs are starting to differentiate^25^ (Fig. 1k,l), but AMPA/kainate receptors are expressed at early timepoints and directly involved in synaptic transmission^36^, we hypothesized that AMPA/kainate receptors are important for early stages of the remyelination process and NMDA receptors for the later stages^13^. To test this hypothesis, we blocked AMPA receptors at a time when OPCs start to differentiate and NMDA receptors start to be expressed. Blocking AMPA receptors at the second week post lesioning did not affect remyelination (Fig. 5e–g; *P*=0.86, Mann–Whitney rank analysis, *n*=4) unlike when blocking AMPA receptors from 3 dpl. These data along with our previous results^13^ indicate that AMPA/kainate receptor activation is essential for the early stages of remyelination, whereas NMDA receptors are important for later stages of remyelination^13^.

**Figure 6:**
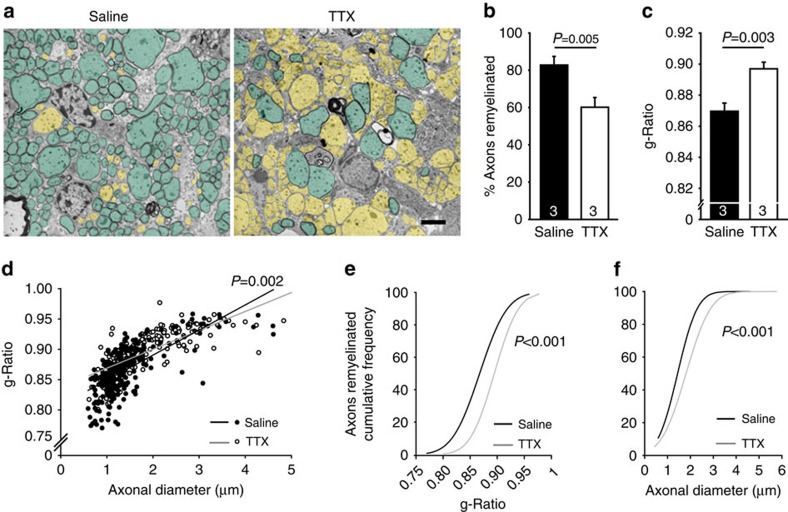
Neuronal activity regulates remyelination. (**a**,**b**) Ultrastructural analysis reveals that fewer axons are remyelinated (green) in lesions treated with TTX (from 3 dpl) compared with saline-treated lesions; unmyelinated axons are coloured in yellow. Scale bar, 1 μm. (**c**,**d**) The g-ratio is larger in TTX-treated samples than in saline-treated lesions. Number of animals are indicated on each bar graph. Data represent mean±s.e.m. *P*-values are from unpaired *t*-test for **b**,**c**. For **d**, each symbol represents an axon and *P*-value from analysis of covariance (ANCOVA). (**e**,**f**) Blocking synaptic input induces a shift in the cumulative frequency distribution of g-ratio and axonal diameter, *P*-values from Kolmogorov–Smirnov test.

Lastly, blocking neuronal activity in the lesion with 50 nM TTX, from 3 dpl, reduced remyelination (Fig. 4e,f,j; *P*=0.03, Mann–Whitney rank analysis, *n*=5) without affecting lesion size (0.64±0.03 mm^2^ (saline) versus 0.48±0.13 mm^2^ (TTX); *P=*0.29, unpaired *t*-test, *n*=5). At the ultrastructural level, it was clear that significantly fewer axons were remyelinated when neuronal activity was blocked (*P=*0.005, unpaired *t*-test, *n*=3; Fig. 6a,b). Blocking neuronal activity with TTX did not affect total axonal number in the lesion (saline: 805161±93822 axons per mm^2^, TTX: 649462±111669 axons per mm^2^; *P=*0.31, unpaired *t*-test, *n*=3), but it did affect remyelination of smaller diameter axons more than larger diameter axons (*P*<0.001, Kolmogorov–Smirnov test, *n*=3; Fig. 6f). In the few axons that were remyelinated when neuronal activity was blocked, the *g*-ratio (the ratio of axon diameter to the outside diameter of the myelin) was higher compared with saline, indicating a thinner myelin sheath (*P=*0.003, unpaired *t*-test, *n*=3; Fig. 6c–e); although the myelin thickness was near identical for larger-diameter axons (axonal diameter ≥2 μm, g-ratio saline: 0.93±0.005, TTX: 0.93±0.002; *P*=0.95, unpaired *t*-test, *n*=3 lesion), it was markedly thinner for small-diameter axons (axonal diameter <2 μm, g-ratio saline: 0.86±0.002, TTX: 0.88±0.002; *P*=4 × 10^−6^, unpaired *t*-test, *n*=3 lesions). Together, this shows that efficient remyelination, especially at smaller-diameter axons, depends on neuronal activity and the synaptic communication between demyelinated axons and OPCs.

### Neuronal activity regulates OPC differentiation

**Figure 7:**
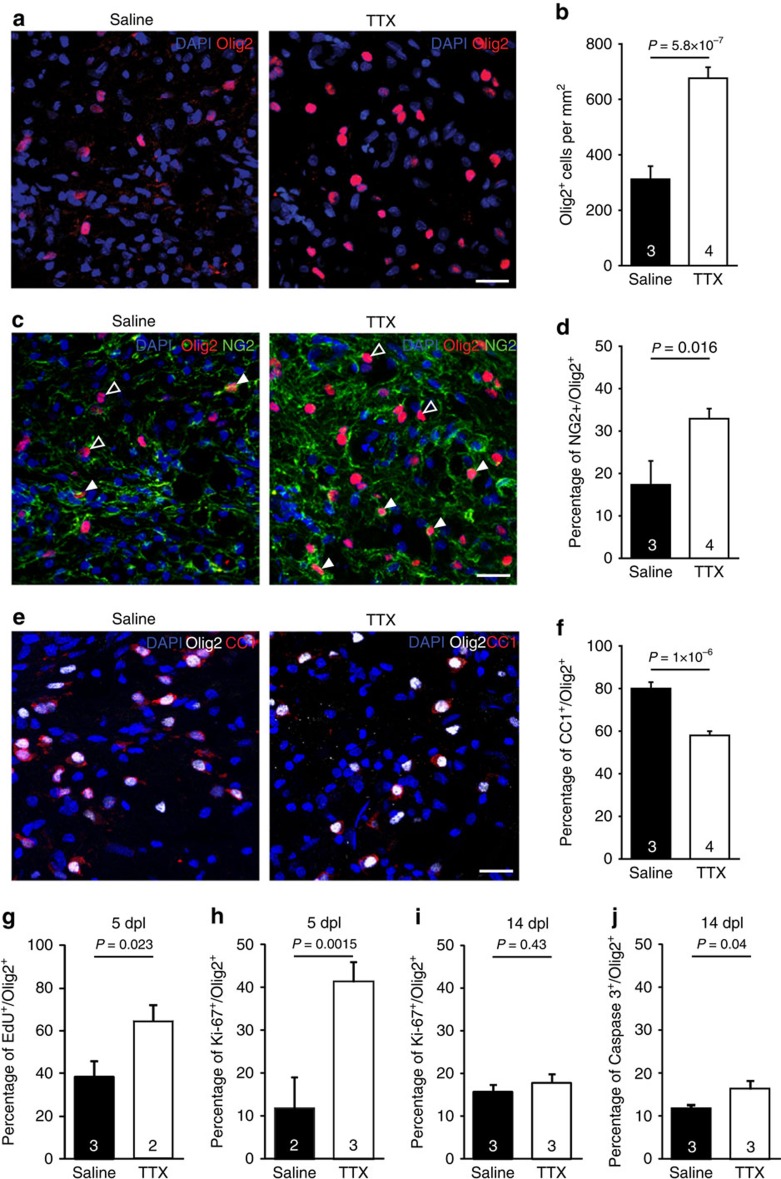
Neuronal activity regulates OPCs differentiation. (**a**,**b**) At the time of differentiation (14 dpl), there was an increase in the number of Olig2^+^ (marking all cells of the oligodendrocyte lineage) cells in TTX-treated lesions (from 3 dpl) compared with saline controls due to (**c**,**d**) a higher proportion of Olig2^+^ cells that were NG2^+^ OPCs (filled arrowheads) and (**e**,**f**) a reduced proportion of Olig2^+^ cells that were CC1^+^ differentiating oligodendrocytes (open arrowheads), indicating that synaptic input is needed for efficient differentiation. (**g**,**h**) At 5 dpl, there are more proliferating Olig2^+^ cells in TTX-treated lesions, as shown by EdU incorporation (**g**) and by Ki-67 staining (**h**). (**i**) No change in proliferation was detected by Ki-67 staining in Olig2^+^ cells between TTX and saline-treated lesions at 14 dpl. However, at 14 dpl there is an (**j**) increase in apoptotic Olig2^+^ cells labelled for caspase 3. All *n* numbers represent animals (three sections per animal) and are indicated on each bar graph. Data are means±s.e.m. *P*-values are from unpaired *t*-test, Welch’s corrected for **d** and **f**. Scale bars, 20 μm.

OPC migration and proliferation can be regulated by neuronal activity^18^,^45^,^46^ and glutamate^12^,^47^,^48^. Therefore, we investigated whether blocking neuronal activity with TTX affected OPC recruitment by staining lesions for NG2 and Olig2. Remarkably, there was a 6-fold increase in the number of OPCs (defined as NG2^+^ and Olig2^+^ cells) in the TTX-treated lesions compared with saline controls (33±9 versus 222±22 cells per mm^2^; *P=*4 × 10^−8^, Welch’s corrected unpaired *t*-test, TTX: *n*=4 and saline: *n*=3) and a 2.7-fold increase in Olig2^+^ cells (Fig. 7a,b; *P*=5.8 × 10^−7^, Welch’s corrected unpaired *t*-test, TTX: *n*=4 and saline: *n*=3), with a higher proportion of Olig2^+^ cells being NG2^+^ OPCs (Fig. 7c,d; *P*=0.016, Welch’s corrected unpaired *t*-test, TTX: *n*=4 and saline: *n*=3) and a lower proportion being CC1^+^ oligodendrocytes (Fig. 7e,f; *P*=1 × 10^−6^, Welch’s corrected unpaired *t*-test, TTX: *n*=4 and saline: *n*=3). The increase in OPCs at 14 dpl is due to a higher proliferation of OPCs at early time points, as we detected nearly 2-fold increase in EdU^+^ Olig2^+^ cells (5-ethynyl-2'-deoxyuridine (EdU) delivered continuously for 3 days; Fig. 7g; *P*=0.023, Welch’s corrected unpaired *t*-test, TTX: *n*=2 and saline: *n*=3) and a 3.5-fold increase in Ki-67^+^ Olig2^+^ cells (Fig. 7h; *P*=1.5 × 10^–3^, Welch’s corrected unpaired *t*-test, TTX: *n*=3 and saline: *n*=2) at 5 dpl, but no difference at 14 dpl (Fig. 7i; *P*=0.43, Welch-corrected unpaired *t*-test, *n*=3). The increase in proliferation of OPCs at early timepoints may reflect the fact that suppressing glutamate release removes a signal that normally limits OPC proliferation^12^,^49^.

Despite a lower proportion of Olig2^+^ cells being CC1^+^ in TTX-treated lesions, there was no difference in the absolute density of CC1^+^ cells between the conditions (*P*=0.25, Welch’s corrected unpaired *t*-test, TTX: *n*=4 and saline: *n*=3). Nevertheless, significantly fewer axons were remyelinated in TTX-treated lesions a week later (*P=*0.005, Welch’s corrected unpaired *t*-test, *n*=3; Fig. 6a,b), indicating that a large proportion of these CC1^+^ oligodendrocytes fail to myelinate. As newly differentiated premyelinating oligodendrocytes depend on axonal signals for survival^50^,^51^, we tested whether neuronal activity affects cell survival by examining the expression of caspase 3, a marker for apoptosis^52^, in Olig2^+^ oligodendrocyte lineage cells. Blocking neuronal activity increased apoptosis by ∼40% compared with saline (Fig. 7j; 12±3% (saline) versus 16±5% (TTX); *P=*0.04, Welch’s corrected unpaired *t*-test, *n*=3) in Olig2^+^ cells. Together, these results indicate that the failure of remyelination that occurs when neuronal activity is blocked is not caused by reduced OPC recruitment but by an increase in apoptosis and impaired differentiation towards myelinating oligodendrocytes.

### Human demyelinated axons upregulate synaptic proteins

**Figure 8:**
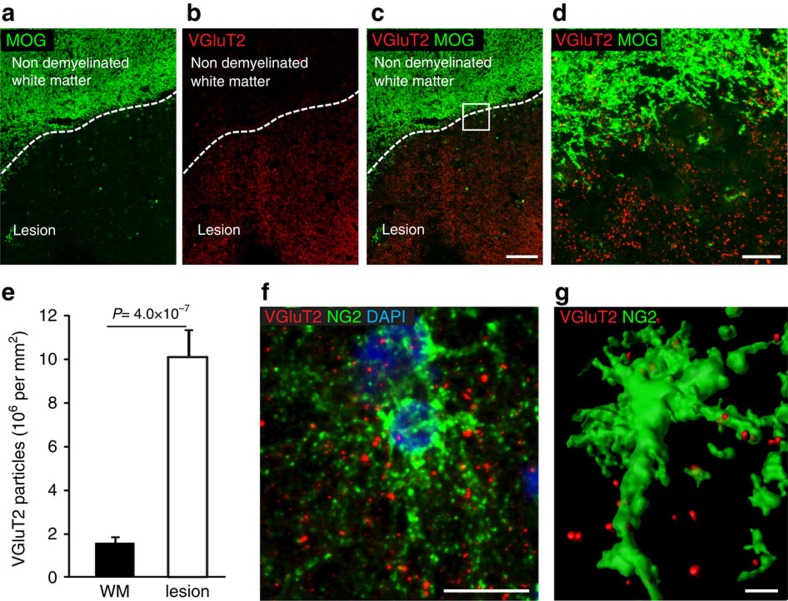
Demyelinated axons in human MS lesions upregulate synaptic proteins. (**a**) MOG staining in a human MS brain tissue sample, clearly demonstrating a demyelinated forebrain white matter lesion. (**b**) VGluT2 is highly expressed in human MS lesions, compared with surrounding normal-appearing white matter. (**c**) Overlay of **a**,**b**. Scale bar, 200 μm. (**d**) Magnified image from **c** of the region indicated by the white box. Scale bar, 50 μm. (**e**) There is a sixfold increase in VGluT2 particle numbers in lesion compared with white matter, *n*=5 patients (one lesion per patient). Data represents mean±s.e.m. *P*-value from unpaired *t*-test. (**f**) VGluT2 puncta are in close proximity to NG2^+^ OPCs. Scale bar, 20 μm. (**g**) Iso-surface 3D deconvolution of an OPC in human MS lesion generated using Imaris software, showing synapses located on the OPC processes. Scale bar, 5 μm.

To determine the significance of our finding for human white matter disease, we examined the expression of VGluT2 in human MS lesions and adjacent normal-appearing white matter (or non-demyelinated white matter). Similar to the rodent demyelinated lesions, there was an approximately sixfold increase in VGluT2 expression in the human MS lesions compared with the normal-appearing white matter (Fig. 8a–e; *P*=4.1 × 10^−7^, unpaired *t*-test, *n*=5 lesions from 5 patients) and the VGluT2 puncta were observed in close proximity of NG2^+^ cells present in the MS lesions (Fig. 8f,g; detected for 150 NG2^+^ cells), with NG2^+^ processes nearly always located near the VGluT2 puncta. Together, these results strongly suggest that, after white matter injury, demyelinated axons establish *de novo* synapses with recruited OPCs in humans.

## Discussion

Remyelination is essential for functional recovery after demyelinating injury. However, spontaneous remyelination often fails, primarily due to failure of OPC differentiation rather than OPC depletion^5^,^53^,^54^. Here we have shown that glutamatergic synaptic transmission between demyelinated axons and OPCs, acting via AMPA receptors, regulates remyelination, and that blocking neuronal activity keeps OPCs in a proliferative stage, preventing them from fully differentiating into myelinating oligodendrocytes. This may explain why remyelination fails in disease.

We found that following demyelination of a CNS white matter tract where all the axons were previously myelinated, recruited OPCs receive synaptic inputs from demyelinated axons. On demyelination, axons increase their expression of the pre-synaptic protein VGluT2 and generate *de novo* synapses with recruited OPCs. We found that a similar mechanism may also occur in human MS lesions. At early timepoints after entering the lesion, the recruited OPCs express mainly AMPA and kainate receptors, presumably at the postsynaptic site. The role of the synaptic input to OPCs seems to be instructing them to remyelinate, as when we infused antagonists specific to distinct stages of synaptic transmission (ω-conotoxin MVIIC to block vesicular release of neurotransmitter, NBQX to block postsynaptic AMPA receptors on the OPCs and TTX to block voltage-gated Na+ channels and thus neuronal activity) into the lesion, all of them impaired remyelination.

Two lines of evidence show that the antagonists are acting locally within the lesion. First, when a dye is infused into a lesion with an osmotic minipump (even with a faster flow rate and larger volume delivered than used in this study) the dye remains within the lesion^41^. Second, no side effects were detected, such as severe disruption of motor function, which would be expected if TTX were to spread into the nearby grey matter beyond the lesion. The lesion model we used is initially devoid of astrocytic cell bodies^24^ and is only occupied by macrophages, OPCs and demyelinated axons (whose cell bodies are distant). Therefore, the antagonist infused into the lesion should be cell specific, as neither OPCs^43^ nor macrophages^44^ express functional N-, Q- and P-voltage-gated calcium channels, blocked by ω-conotoxin MVIIC. Similarly, macrophages in the lesion do not respond to glutamate and demyelinated axons have not been reported to express AMPA receptors (nor did we detect glutamate evoked calcium changes in demyelinated axons); thus, NBQX infusion into the lesion should only affect the AMPA receptors on OPCs. Furthermore, as administrating NBQX later, at the time when astrocytes have reappeared in the lesion^24^, did not affect remyelination, we can attribute the effect of NBQX administration to an effect on OPC AMPA receptors. By combining three distinct antagonists of synaptic transmission, all of which give the same results, we were able to demonstrate a role for synaptic communication between axons and OPCs in regulating remyelination *in vivo*.

Despite our demonstration that NBQX blocks remyelination, paradoxically, a systemic administration of NBQX to EAE mice was found to ameliorate the clinical severity of the disease^21^,^22^. This may reflect neuroprotection from glutamate excitotoxicity induced by the severe inflammation occurring in EAE lesions^7^,^55^. In contrast, we used local administration of NBQX into the white matter of a model that fully remyelinates without significant axonal loss, thereby identifying a mechanism of white matter regeneration rather than of white matter damage.

Blocking neuronal activity with TTX, which blocks voltage-gated Na^+^ channels, reduced remyelination in a similar manner to blocking vesicular release of neurotransmitter with ω-conotoxin MVIIC or to blocking with NBQX the AMPA/kainate receptors, located postsynaptically at the axon-OPC synapse^36^. This resulted in fewer axons being remyelinated, with smaller diameter axons being more affected, just as blocking vesicular release of neurotransmitter preferentially affects myelination of small-diameter axons^16^. We assume that the effects of TTX are predominantly to block axonal action potentials. However, OPCs also express voltage-gated Na^+^ channels and it is currently unknown what role they have in OPC biology. Although, we cannot exclude an effect of TTX on voltage-gated Na^+^ channels in OPCs, activation of OPC Na^+^ channels requires the membrane to become depolarized to ∼−40 mV, which is unlikely to occur without activity-driven input.

The lack of activity-driven signals in the TTX-treated samples caused an up to 3.5-fold increase in proliferation of OPCs at 5 dpl, resulting in a ∼6-fold increase in NG2- and Olig2-positive cells at 14 dpl, indicating that blocking neuronal activity predominantly keeps OPCs in a proliferative stage and leads to a lower proportion of differentiated oligodendrocytes. However, the absolute numbers of differentiated oligodendrocytes (CC1^+^) at 14 dpl remain the same. Intriguingly, despite equal numbers there is less remyelination. In our lesion model we see an overproduction of CC1^+^ cells at 14 dpl that presumably do not reach targets, as there are fewer CC1^+^ cells at 21 dpl, comparable to early development where there is an overproduction of oligodendrocytes, many of which die^31^. This possibly reflects a spontaneous differentiation of OPCs, which occurs at high OPC density^56^. Thus, a higher proportion of untargeted differentiation, owing to higher OPC density, could explain why equal numbers of CC1^+^ cells still lead to impaired remyelination in TTX-treated lesions.

The effect of blocking the activity of the demyelinated axons is likely to be mediated via synaptic input and glutamate release, as blocking vesicular release and AMPA receptors on OPCs also resulted in impaired remyelination. Previously, we showed remyelination depended on activation of NMDA receptors^13^, but NMDA-evoked currents in OPCs only appear at the second week post lesion induction, which suggests that NMDA receptors only play a role at later stages of remyelination. Conversely, AMPA/kainate receptors are expressed throughout the regenerative process^57^ and activation of AMPA receptors during the period of proliferation is essential for remyelination, whereas activation of AMPA receptors later, at the onset of differentiation (a time when NMDA-evoked currents start to be detected), is not. Thus, using this remyelination model we have revealed a potential distinct role for different glutamate receptors expressed on OPCs. Presumably, glutamate released at the axon-OPC synapse activates the AMPA receptors directing OPCs towards the axon to myelinate, as synaptic inputs are important for axonal selection^15^,^16^. Once the axon-OPC contact is made, NMDA receptors (as they tend to be expressed on the processes^6^,^58^) become important to direct myelination^13^. Our data therefore suggest that neuronal activity fine-tunes OPC proliferation and times the onset of OPC differentiation by acting on AMPA receptors and regulates remyelination by acting on NMDA receptors. Thus, when OPCs do not receive synaptic inputs from active demyelinated axons they remain in a proliferative state, because they lack their inductive signal to differentiate.

Remyelination efficiency decreases with age, as a result of a failure of differentiation^59^,^60^ rather than OPC depletion^5^,^59^,^61^. Our data indicate that for remyelination to occur the demyelinated axons must recapitulate early development and direct OPC differentiation by maintaining electrical activity to release glutamate onto OPCs. In fact, on demyelination, axons switch their isoform of voltage-gated sodium channels (Na_v_) from Na_v_1.6 (found at the nodes of myelinated axons^62^) back to Na_v_1.2 (ref. 63) (found in unmyelinated axons during development^62^). Furthermore, in active MS lesions (many of which undergo remyelination), axons express Na_v_1.2 channels^64^,^65^ and upregulate synaptic proteins^19^, whereas axons in chronic lesions that have failed to remyelinate do not^64^. This, together with our results, suggests a commonality of response by injured axons: the demyelinated axons undergo a similar response to that of transected peripheral axons, which by reactivating silent growth programmes revert to a developmental state for efficient regeneration^66^. Our results indicate that any perturbation of signals that induce demyelinated axons to remain active and/or regain synaptic contacts with recruited OPCs will result in a failure of OPC differentiation and repair. Consistent with our data, OPCs are present in chronic lesions^5^, presumably lacking an inductive signal from the demyelinated axons for differentiation. Hence, it is conceivable that the use of agonists that promote impulse propagation, vesicular release and/or AMPA receptor currents in OPCs may promote OPC differentiation and myelin repair in disease. Thus, our results reveal a missing link for understanding myelin regeneration and open up new and pharmacologically tractable avenues by which the arrest of OPC differentiation in chronic MS lesions could be therapeutically reversed.

## Methods

### Toxin-induced focal demyelination

Female Sprague–Dawley rats aged 10–14 weeks of age (190–250 g) were used. Experiments were performed in compliance with UK Home Office regulations. In this toxin model, remyelination proceeds to completion (between 3 and 4 weeks after induction of the lesion) with a clear temporal separation between the acute demyelination phase and the subsequent stages of remyelination: OPC recruitment (3–10 dpl), differentiation (10–14 dpl) and remyelination (21–28 dpl)^25^. For electrophysiological experiments, focal demyelination was induced bilaterally by injection of EB into the CCP^24^ and animals were culled by decapitation. Briefly, anaesthesia was induced (4%) and maintained (1.5%–2.5%) with isoflurane (Henry Schein, Animal Health, Dumfries, UK) and the animal placed in a stereotaxic frame. After incision of the skin, holes were drilled at stereotaxically defined locations, then a Hamilton syringe lowered in place to inject 4.0 μl of 0.01% EB (w/v) in 0.9% saline (Vetivex) at 1 μl min^−1^. The skin was sutured using Vicryl 5.0 (Henry Schein). Analgesia was provided by injection of buprenorphine (0.005 mg per 100 g body weight, Vetergesic) before surgery and orally (1 g 10% Vetergesic in flavoured jelly per 100 g body weight) twice a day, up to 2 days until full recovery. Animals were monitored twice daily until full recovery. For continuous local delivery, unilateral lesions were performed and blockers (Tocris), dissolved in 0.9% saline, were infused into the demyelinated lesion by an osmotic minipump, with a reservoir volume of 100 μl and a flow rate of 0.11 μl h^−1^ (Alzet Micro-Osmotic Pumps, model 1004, DURECT Corporation), which was attached through a vinyl tube spacer (Plastics One Inc., Roanoke, Virginia) to a 30-gauge (6.5 mm) cannula implanted just above the lesion. The minipump was placed subcutaneously and the cannula was fixed to the skull with cyanoacrylate gel adhesive (applied under the base of the cannula head before insertion), as well as two anchoring screws and dental acrylic cement (a 1:1 volume mix of Palacos MG+G powder and liquid, Heraeus Kulzer)^13^. Drug delivery into the lesion did not occur until 3 dpl (the start of the OPC recruitment stage^25^; Fig. 1l) or at 10 dpl (at the start of OPC differentiation^25^; Fig. 1l). The onset of drug delivery into the lesion was controlled by varying the length of the tube that connects the minipump to the cannula and by inserting a sterile corn oil drop (Sigma) to prevent diffusion of the drug solution into the initial saline solution. For proliferation studies at 5 dpl, EdU was administered by osmotic minipump, dissolved as 5 μg μl^−1^ of saline solution, from 3 dpl. We have previously shown that the delivery is local with no significant dye diffusion detectable outside the lesion^41^. Rats were randomly assigned to treatment (ω-conotoxin MVIIC, 11 μM; NBQX, 250 μM; TTX, 50 nM) or control groups (0.9% saline infusion). Samples were included in the analysis only if the cannula was found within 200 μm of the lesion. For remyelination studies, animals were perfuse fixed under terminal anaesthesia (pentobarbitone sodium) 3 weeks after lesion induction.

### Acute brain slices for electrophysiology and Ca^2+^ imaging

For the majority of the experiments, rat sagittal slices (225 μm for patch clamping and Ca^2+^ imaging, 300 μm for compound action potential) including the CCP containing the lesion were made, either between 5 and 10 dpl, when OPC numbers within the lesion peak, or at 21 dpl, when remyelination is near completion^25^. For the recordings of synaptic input in normal adult white matter at P5–P7 and P98–P105 (3 months old), slices were obtained from NG2-EYFP knock-in male and female mice^67^ with the same procedure. Slices were made in an ice-cold bicarbonate-buffered (5% CO_2_/95% O_2_, pH 7.4) slicing solution containing (mM): 120 NaCl, 26 NaHCO_3_, 1 NaH_2_PO_4_, 2.5 KCl, 2 MgCl_2_, 2 CaCl_2_, 10 glucose and 1 kynurenic acid (a broad-spectrum glutamate receptor antagonist). For all experiments, slices were superfused at 22±1 °C with HEPES-buffered solution containing (mM): 144 NaCl, 2.5 KCl, 10 HEPES, 1 NaH_2_PO_4_, 2.5 CaCl_2_, 10 glucose, 0.1 glycine (to co-activate NMDA receptors), 0.005 strychnine (to block glycine receptors), pH adjusted to 7.4 with NaOH and bubbled with 100% O_2_.

### Whole-cell patch clamp recordings

Whole-cell patch clamp recordings were conducted using Multiclamp 700B (Molecular Devices) connected to a 16-bit analogue-to-digital converter (Digidata 1440A, Molecular Devices). The pClamp 10 software package was used for data acquisition (sampling rate 50 kHz, filter 10 kHz) and analysis. Electrodes contained solution comprising (mM): 130 Cs-gluconate, 4 NaCl, 0.5 CaCl_2_, 10 HEPES, 10 BAPTA, 4 MgATP, 0.5 Na_2_GTP, 2 K-Lucifer yellow, pH set to 7.3 with CsOH, 295 mOsm. Series resistance was 5–25 MΩ (if higher, cells were excluded from the analysis) and electrode junction potentials (−14 mV) were corrected for. Cells were identified (Fig. 2a,b and Supplementary Fig. 2) by their post-recording dye-fill morphology^8^,^68^ and confirmed by antibody labelling against proteoglycan NG2 (11/11) and the transcription factor Olig2 to identify oligodendrocyte progenitors (16/16). OPCs had a steady-state input resistance of ∼900 MΩ and a low cell capacitance of 31.6±3 pF. OPCs were defined both by morphology and in some cases by labelling with antibodies against NG2 and Olig2, and were electrophysiologically and morphologically distinct from the abundant macrophages in 5–10 dpl lesions (Supplementary Fig. 2).

### Synaptic current analysis

A synaptic current was defined to occur if its amplitude was >3 times the s.d. of the baseline current noise and its 10%–90% decay time was longer than its rise time. Events were recorded using a Minidigi 1B (Molecular Devices, sampling 1 kHz), then detected and analysed with WinEDRv3.2.6 and WinWCP v4.3.7 (University of Strathclyde).

### Compound action potential recordings

Stimulation was delivered by a voltage stimulator (Digitimer) through tungsten bipolar electrodes (WPI) and recorded with a glass electrode (0.8–1 MΩ) filled with external HEPES-buffered solution connected to an amplifier (Multiclamp 700B). At the end of each experiment, different lipophilic dyes (DiD and DiI, Molecular Probes) were applied to mark the stimulation (DiD) and recording (DiI) electrode location (Fig. 1c). The distance between the electrodes was measured using Image J.

### Intracellular Ca^2+^ imaging

Slices were incubated for 1 h at 22 °C in slicing solution with 10 μM Fluo4-AM, 25 μM FuraRed-AM and 0.25% pluronic F-127 (Life Technologies), followed by 1 h de-esterification. Fluorescence was excited at 488 nm by scanning laser (Leica Sp5, × 40 water-immersion objective with 0.80 numerical aperture) and emitted light was collected at 489–583 nm for Fluo4 and at 622–703 nm for FuraRed. The ratio of the emission intensities of Fluo4 (maximum emission when bound to Ca^2+^) and FuraRed (maximum emission when Ca^2+^ is free) was used to measure the increase in [Ca^2+^]_i_ and only ratios that reflected both an increase of fluorescence emitted at 489–583 nm and a decrease in fluorescence emitted at 622–703 nm were used. After imaging, three morphologically distinct cells were dye-filled via patch pipette for *post-hoc* identification of the area imaged, and to allow for identification of the cell types within the imaged area with immunohistochemistry (Supplementary Fig. 3a–c).

### Immunohistochemistry

Fixed slices were incubated for 5 h in 0.5% Triton X-100, 10% goat serum in PBS at 21 °C, then with primary antibody at 21 °C overnight, and then for 5 h at 21 °C with secondary antibody; both antibody incubations were followed by three 20-min washing steps in PBS. Cryostat sections were incubated for 1 h in 0.1% Triton X-100, 10% goat or donkey serum in PBS at 21 °C, then with primary antibody at 4 °C overnight (apart from goat Olig2, which was incubated for 5 h at 21 °C), and then for 1 h at 21 °C with secondary antibody. All antibodies (primary and secondary) used have been previously used and validated in our laboratories^13^,^57^,^69^. Primary antibodies were as follows: rabbit caspase-3 (Millipore: AB3623, 1:10), rabbit GFAP (DAKO: Z0334, 1:200), guinea pig NG2 (a kind gift from W.B. Stallcup: 1:400), rabbit NG2 (Millipore: AB5320, 1:300), mouse NG2 (Millipore: MAB5384, 1:250), rabbit Olig2 (Millipore: AB9610, 1:300), goat Olig2 (R&D Systems: AF2418, 1:200), guinea pig VGluT2 (Synaptic System: 135 404, 1:500), mouse neurofilament 160/200 (Sigma: N2912, 1:200), mouse CC1 (Calbiochem: OP80, 1:200), rabbit ki-67 (Abcam: AB16667, 1:300) and mouse CD11b (Serotec: MCA711, 1:100). 4,6-diamidino-2-phenylindole (10 min, D9542, Sigma, 1 μg ml^−1^) was used to label nuclei. Secondary antibodies (goat) were for rabbit IgG (Life Technologies: A11034, A11036 and A21245, 1:500), guinea pig IgG (Life Technologies: A21435, 1:500) and mouse IgG (Life Technologies: A11029, A11031 and A21236, 1:500). Secondary antibodies (donkey) were for goat IgG (Sigma: SAB4600032, 1:500) and rabbit IgG (Life Technologies: A-31573, 1:500). Samples to be immunolabelled with GFAP and Ki-67 underwent antigen retrieval, by incubation at 80 °C for 20 min in citrate buffer (10 mM sodium citrate, 0.05% Tween 20, pH 6.0), before the immunohistochemistry procedure outlined above. EdU was detected using a Click-iT EdU Alexa Fluor 555 Imaging Kit (Life Technologies, C10338), according to the manufacturer’s instructions. Briefly, antigen retrieval was carried out as described, sections were permeabilized for 1 h with 0.05% Triton in PBS, washed for 15 min in 2% BSA in PBS, then Click-iT reaction cocktail applied for 30 min, sections were washed again with 2% BSA, then the immunohistochemistry procedure continued as described above. Coverslips were mounted with Fluoromount-G. For quantitative analysis, the whole lesion (three sections with lesions per animal) was photographed and each picture given a number. Random numbers were generated to select the pictures to be analysed blindly (4 to 6 pictures per animal, minimum 12 pictures for each condition).

### Human tissue immunohistochemistry

Postmortem MS tissues were obtained from the UK MS Society Tissue Bank at Imperial College and were collected via a prospective donor scheme following fully informed consent (08/MRE09/31). The demographic data and clinical characteristics are shown in Supplementary Table 1. The clinical diagnosis of MS was confirmed based on the patient history and a detailed neuropathological analysis. The presence of lesions was confirmed as described previously^70^. Ten-micrometre sections from snap-frozen human tissue blocks were permeabilized with methanol at −20 °C for 15 min, sections were incubated for 1 h in 0.1% Triton X-100, 10% goat serum in PBS at 21 °C, then with primary antibody at 4 °C overnight, and then for 2 h at 21 °C with secondary antibody. Primary antibodies were rabbit NG2 (as above, 1:100), guinea pig VGluT2 (as above, 1:500), rabbit MOG (1:10, in house) and mouse neurofilament (as above, 1:1,000), and all secondary antibodies as above. 4,6-diamidino-2-phenylindole (10 min, Sigma, 100 mg ml^−1^) was used to label nuclei and autofluorescence quenched with 0.1% Sudan Black for 30 min. Coverslips were mounted with Vectashield. All images were 5 μm *z*-stacks taken at 0.5 μm intervals on a Leica EL6000 confocal microscope. For VGluT2 quantification, white matter lesions were identified using a sequential section stained for MOG by immunohistochemistry. Lesion edges were defined by the abrupt absence of MOG^+^ myelin in anatomically aligned tracts with continuing axons. Sections were aligned and white matter lesions outlined on fluorescently stained slides. For each MS case, one white matter lesion that contained a high number of NG2^+^ cells was analysed. The presence of axons within the lesion was confirmed by neurofilament staining. For quantitative analysis, all images were taken at × 63 at the same gain, resolution and exposure settings within lesions and adjacent areas of normal-appearing white matter based on the MOG staining. A further sequential section was stained with the same protocol as above for MOG (1:10, in house) and VGluT2 (1:500), to confirm lesion localization. After acquisition, all images were analysed blindly (three images per lesion and one lesion per patient), by a different person to the one who had acquired the images.

### Histological analysis of demyelination and remyelination

Animals were perfused with 4% glutaraldehyde (in phosphate buffer with 0.72 mM CaCl_2_) and the brains left to post fix for 4–7 days. Tissue blocks encompassing the CCP were cut^24^. Blocks were processed through 2% osmium tetroxide (Oxkem Ltd), dehydrated in increasing concentration of ethanol and embedded in resin (TAAB Laboratories). One-micrometre sections were cut using a Leica microtome (RM 2065) and stained with Toluidine blue. In these sections, remyelinated axons can be easily distinguished from normally myelinated axons outside the lesion by the thinness of the myelin sheath and remyelinated axons can be distinguished from demyelinated axons (Supplementary Fig. 1). Remyelination was independently ranked blindly by three persons.

### Electron microscopy

Samples used for histological analysis were further processed for electron microscopy. Resin blocks were cut in 90-nm sections on an ultramicrotome (Reichert Ultracut E) with a diamond knife (Diatome) and visualized using a transmission electron microscope (Hitachi H600). Random images (saline *n*=10 and TTX *n*=12) from the lesions were taken and developed on Electron Microscope Film 4489 (Kodak), then scanned at high resolution (12,800 dpi × 12,800 dpi). The number of axons remyelinated and their g-ratios (the ratio of axon diameter to the outside diameter of the myelin) were analysed with ImageJ software (version 1.45 s) by blinded analysis. A total of 302 (TTX) and 312 (saline) axons were analysed.

### Statistics

Numbers of experiments are indicated on bars. Data are presented as mean±s.e.m., unless stated otherwise. When relevant, normality of data was assessed using Shapiro–Wilk tests. Non-parametric tests that do not assume data follow a normal distribution gave the same conclusions for significant and nonsignificant differences in all cases. One-way ANOVA followed by Holm–Bonferroni-corrected *post-hoc t*-tests were used to compare multiple samples. Other *P*-values for comparison of means are from Student’s two-tailed *t*-tests, with Welch’s correction when variances were unequal. Ranking was analysed with Mann–Whitney ranking tests. Analysis of covariance was used to analyse regression lines. Cumulative frequency was analysed with Kolmogorov–Smirnov tests.

## Additional information

**How to cite this article:** Gautier, H. O. B. *et al*. Neuronal activity regulates remyelination via glutamate signalling to oligodendrocyte progenitors. *Nat. Commun*. 6:8518 doi: 10.1038/ncomms9518 (2015).

## Supplementary Material

Supplementary Information Supplementary Figures 1-3 and Supplementary Table 1
