# Supplementary material for: Neuronal activity regulates remyelination via glutamate signalling to oligodendrocyte progenitors
Source: Nat Commun. 2015 Oct 6;6:8518. doi: 10.1038/ncomms9518 (PMC4600759; doi:10.1038/ncomms9518)
Supplement: Supplementary Information — Supplementary Figures 1-3 and Supplementary Table 1 [file ncomms9518-s1.pdf]

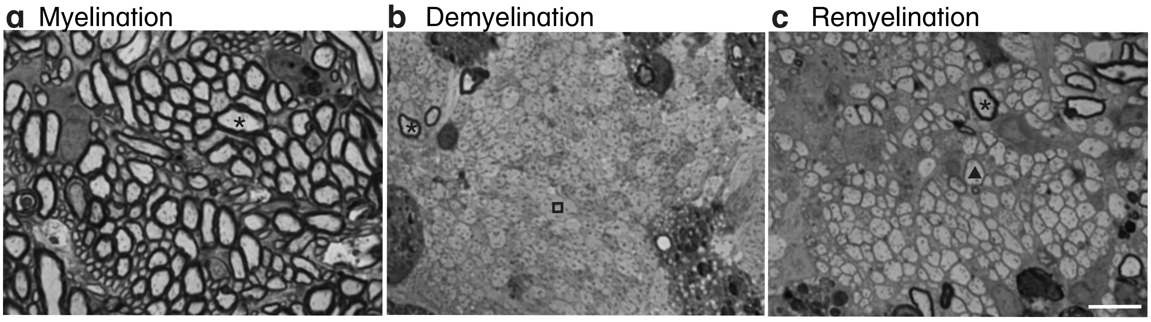

Supplementary Figure 1.
Ethidium bromide toxin model of remyelination. (**a**) The adult CCP is a near fully myelinated white matter tract. (**b**) After the injection of 0.01% EB, demyelination occurs with little to no axonal pathology and (**c**) 21 days after the injection of EB remyelination is proceeding to completion. Symbols show: * myelinated axons, ¤ demyelinated axon, ▲ remyelinated axon. All sections are toluidine blue stained 1μm resin sections. Scale bar, 10 μm.

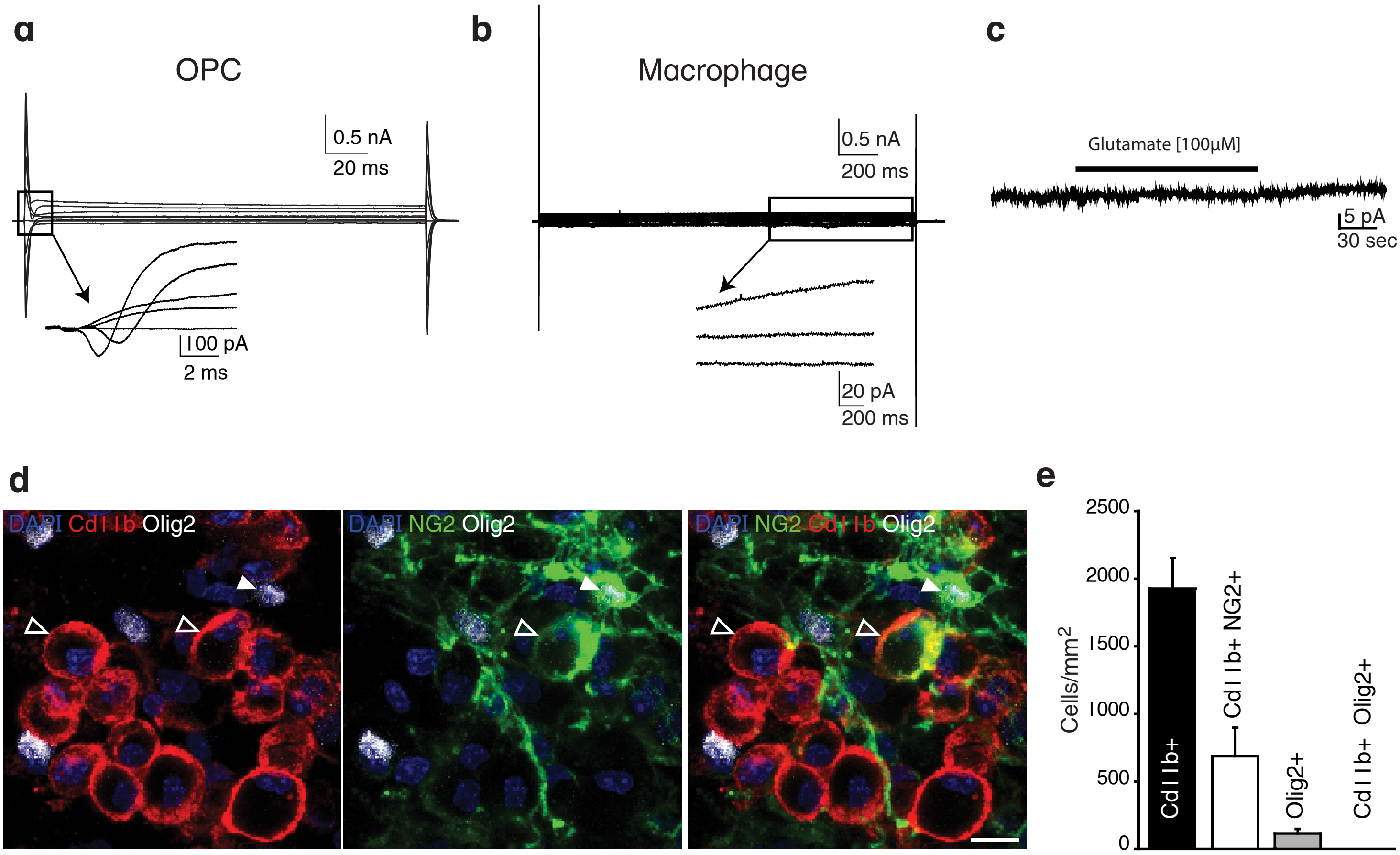

Supplementary Figure 2.
(**a** and **b**). Representative current-voltage relationship for (**a**) an OPC with voltage-gated sodium current and (**b**) macrophages, with a characteristic outward current at depolarized potentials; (voltage steps **a** from -134 to 6 mV, 200 ms duration and **b** from -134 mV to 46 mV, 500 ms). (**a**) Inset shows leak subtracted traces of the first 5 ms. (**c**) No glutamate evoked current is detected in whole-cell voltage-clamped macrophages. (**d**) Macrophages (Cd11b$^+$) have a very distinctive circular morphology (open arrowhead) compared to OPCs (Olig2$^+$NG2$^+$, filled arrowhead). (**e**) Although 40% of Cd11b$^+$ cells are also NG2$^+$, none are Olig2$^+$. Data represent means +/- s.e.m., scale bar, 20 µm.

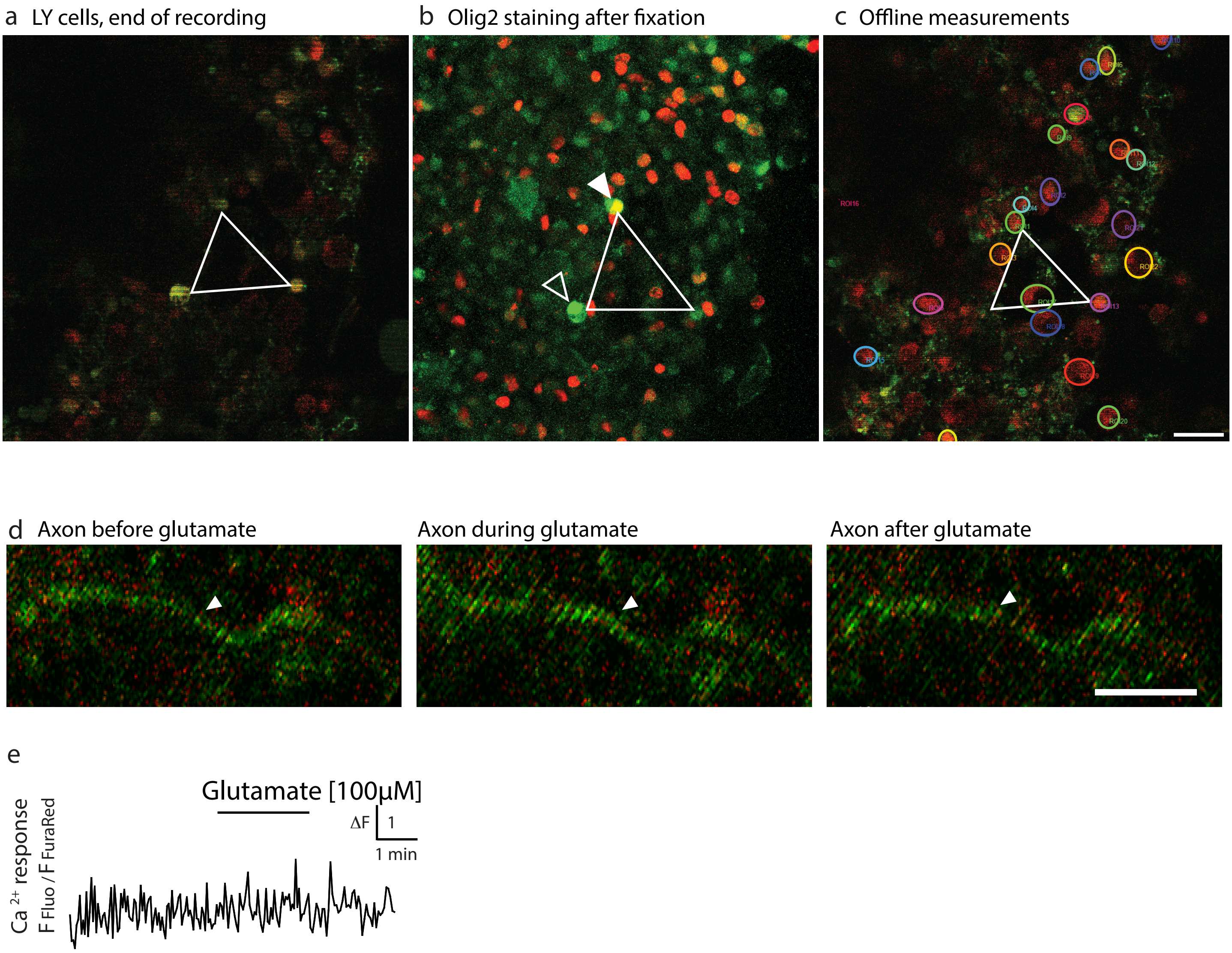

Supplementary Figure 3.
(a) At the end of the Ca^{2+} imaging, three cells were filled with LY, to clearly mark the field of view. After fixation and Olig2 staining (b), the LY cells were used as reference points to determine the orientation of the field of view and allow identification of the imaged cells (c). Scale bar, 25 µm.
(d and e) No glutamate evoke $[Ca^{2+}]_i$ changes were detetected in demyelinated axons (arrowhead), measured by taking the fluorescent intensity ratio of Fluo-4/FuraRed. Scale bar, 10 µm.

Supplementary Table 1 Clinical characteristics of MS cases

**MS case**	**Type**	**Age (yrs)**	**Gender**	**Duration (yrs)**	**PM delay (hrs)**
MS402	SPMS	46	M	20	12
MS497	SPMS	60	F	29	26
MS408	SPMS	39	M	10	21
MS422	SPMS	58	M	13	25
MS438	SPMS	53	F	18	17
